# The m6A reader YTHDC1 regulates muscle stem cell proliferation via PI4K–Akt–mTOR signalling

**DOI:** 10.1111/cpr.13410

**Published:** 2023-02-01

**Authors:** Jin Liu, Hongna Zuo, Ziliu Wang, Wei Wang, Xuezhen Qian, Yingyuan Xie, Di Peng, Yubin Xie, Liquan Hong, Wanling You, Huiling Lou, Guanzheng Luo, Jian Ren, Bin Shen, Jinping Zheng, Hu Wang, Zhenyu Ju

**Affiliations:** ^1^ Key Laboratory of Regenerative Medicine of Ministry of Education Institute of Aging and Regenerative Medicine, Jinan University Guangzhou China; ^2^ State Key Laboratory of Reproductive Medicine, Gusu School, Women's Hospital of Nanjing Medical University, Nanjing Maternity and Child Health Care Hospital Nanjing Medical University Nanjing China; ^3^ MOE Key Laboratory of Gene Function and Regulation, Guangdong Province Key Laboratory of Pharmaceutical Functional Genes, State Key Laboratory of Biocontrol, School of Life Sciences Sun Yat‐sen University Guangzhou China; ^4^ State Key Laboratory of Oncology in South China, Cancer Center, Collaborative Innovation Center for Cancer Medicine, School of Life Sciences Sun Yat‐sen University Guangzhou China; ^5^ Department of Clinical Laboratory Affiliated Hospital of Hangzhou Normal University Hangzhou China; ^6^ Department of Geriatrics, National Key Clinical Specialty, Guangzhou First People's Hospital, School of Medicine South China University of Technology Guangzhou China; ^7^ Department of Public Health and Preventive Medicine Changzhi Medical College Changzhi P. R. China; ^8^ Key Laboratory of Aging and Cancer Biology of Zhejiang Province, School of Basic Medical Sciences Institute of Aging Research, Hangzhou Normal University Hangzhou China

## Abstract

Muscle stem cells are required for the homeostasis and regeneration of mammalian skeletal muscles. It has been reported that RNA N6‐methyladenosine (m6A) modifications play a pivotal role in muscle development and regeneration. Nevertheless, we know little about which m6A reader regulates mammalian muscle stem cells. Here, we discovered that the m6A reader Ythdc1 is indispensable for mouse skeletal muscle regeneration and proliferation of muscle stem cells. In the absence of Ythdc1, Muscle stem cells in adult mice are unable to exit from quiescence. Mechanistically, Ythdc1 binds to m6A‐modified Pi4k2a and Pi4kb mRNAs to regulate their alternative splicing and thus PI4K–Akt–mTOR signalling. Ythdc1‐null muscle stem cells show a deficiency in phosphatidylinositol (PI) 3,4,5‐trisphosphate, phospho‐Akt and phospho‐S6, which correlates with a failure in exit from quiescence. Our findings connect dynamic RNA methylation to the regulation of PI4K–Akt–mTOR signalling during stem cell proliferation and adult tissue regeneration.

## Graphical abstract

YTHDC1 regulated alternative splicing and promoted intron retention in satellite cells (SCs). It would interact with N6‐methyladenosine‐enriched Pi4kb and Pi4k2a to regulate their alternative splicing which have an effect on the production of phosphatidylinositol (PI) 3,4,5‐trisphosphate (PIP3). Activation of AKT–mTOR was interfered which resulted from lower content of PIP3 in YTHDC1‐null SCs compared with that in wild‐type SCs. As the requirement of AKT–mTOR for muscle stem cells activation, muscle stem cells that deplete YTHDC1 would stay in quiescence. Taken together, we uncovered that YTHDC1 controls SCs activation via the PIP3–AKT–mTOR axis.

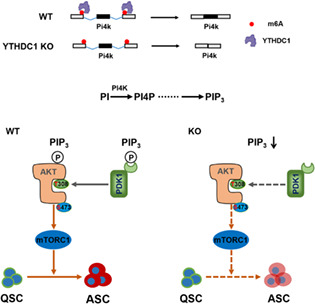

## 1 INTRODUCTION

Skeletal muscle stem cells, also known as satellite cells (SCs), are responsible for maintenance and repair of muscle tissue throughout lifespan.^1^ Muscle stem cells are located between sarcolemma of myofibers and the surrounding basal lamina in muscle tissue.^2^ Adult SCs which remain mitotically quiescent under normal condition characteristically express a paired box transcription factor Pax7.^3^ Responding to exercise and injury, the quiescent SCs is first activated and then enter cell cycle to proliferate which results in an increase in number.^4^ Following proliferation, the offspring of SCs differentiate and fuse to form multinucleated myotubes or fuse to damaged myofibers to repair injured muscle tissue.^4^, ^5^ Besides, a minority of the proliferating SCs return to dormant state to replenish the stem cell pool.^5^ It was documented that SCs fate is governed by a number of intrinsic and extrinsic signals including Notch, Wnt, mTOR, p38/MAPK, insulin‐like growth factor, fibroblast growth factor (FGF) and others.^6^, ^7^, ^8^, ^9^, ^10^, ^11^ However, regulation of SCs fate is not fully understood.

Dynamic and reversible N6‐methyladenosine (m6A) is one of the most prominent post‐transcriptional modifications on the RNA molecule in eukaryotic cells.^12^ The m6A modification is catalysed by a methyltransferase complex consisting of Mettl3 and other cofactors (writer).^12^ The reversible RNA modification can be erased by demethylases, Fto and Alkbh5 (eraser).^12^ Readers of m6A‐modified RNA (reader) preferentially bind to target mRNA to control cellular fate of modified mRNA, including gene expression, mRNA stability, mRNA translation, splicing and nuclear export of mRNA.^13^ Similar to epigenetic modifications, m6A methylation of mRNA is involved in a variety of biological processes such as cell expansion, cell differentiation, cell apoptosis and cell migration and invasion.^14^

A number of studies showed that m6A modification is important for cell fate decision of somatic stem cells and embryonic stem cells, maintenance and development of cancer stem cells.^15^, ^16^, ^17^, ^18^, ^19^, ^20^, ^21^ In mouse skeletal muscle, the global levels of m6A of regenerating muscle tissue decline during the transition of SCs from proliferation to differentiation.^22^ Mettl3 regulates cell differentiation of C2C12 myoblast.^22^, ^23^ In SCs‐specific *Mettl3* knockout (KO) mice, regenerative capacity of skeletal muscle is impaired severely due to the failure in quiescence exit of SCs.^24^ Fto is demonstrated to be involved in myogenesis which depends on its demethylase activity.^25^ All of these research results indicate that m6A plays a critical role in myogenesis of SCs and myoblast.

Ythdc1 is an identified reader of m6A‐modified RNA which is localized in the nucleus.^26^ Depletion of Ythdc1 in mice leads to embryonic lethality. It was documented that Ythdc1 is involved in mRNA splicing, polyadenylation, nuclear export and degradation.^27^, ^28^, ^29^ It plays a pivotal role in mouse oocyte development, maintenance of mouse ES cells, normal and malignant haematopoiesis in a m6A‐dependent manner.^27^, ^29^, ^30^, ^31^ However, we know little about the role of Ythdc1 in SCs. In this study, we uncover that Ythdc1 is essential for the SCs proliferation in adult mice. Ythdc1 regulates alternative RNA splicing of Pi4kb and Pi4k2a that are critical enzyme to generate phosphatidylinositol (PI). In Ythdc1‐null SCs, activation of Akt–mTOR is interrupted which results from inadequate PI 3,4,5‐trisphosphate (PIP3). Akt–mTOR pathway is required for SCs proliferation, thus SCs that Ythdc1 is deleted are unable to exit from dormancy to enter cell cycle.

## 2 RESULTS

### 2.1 Depletion of Ythdc1 in SCs impairs regenerative capacity of muscle in adult mice

**FIGURE 1:**
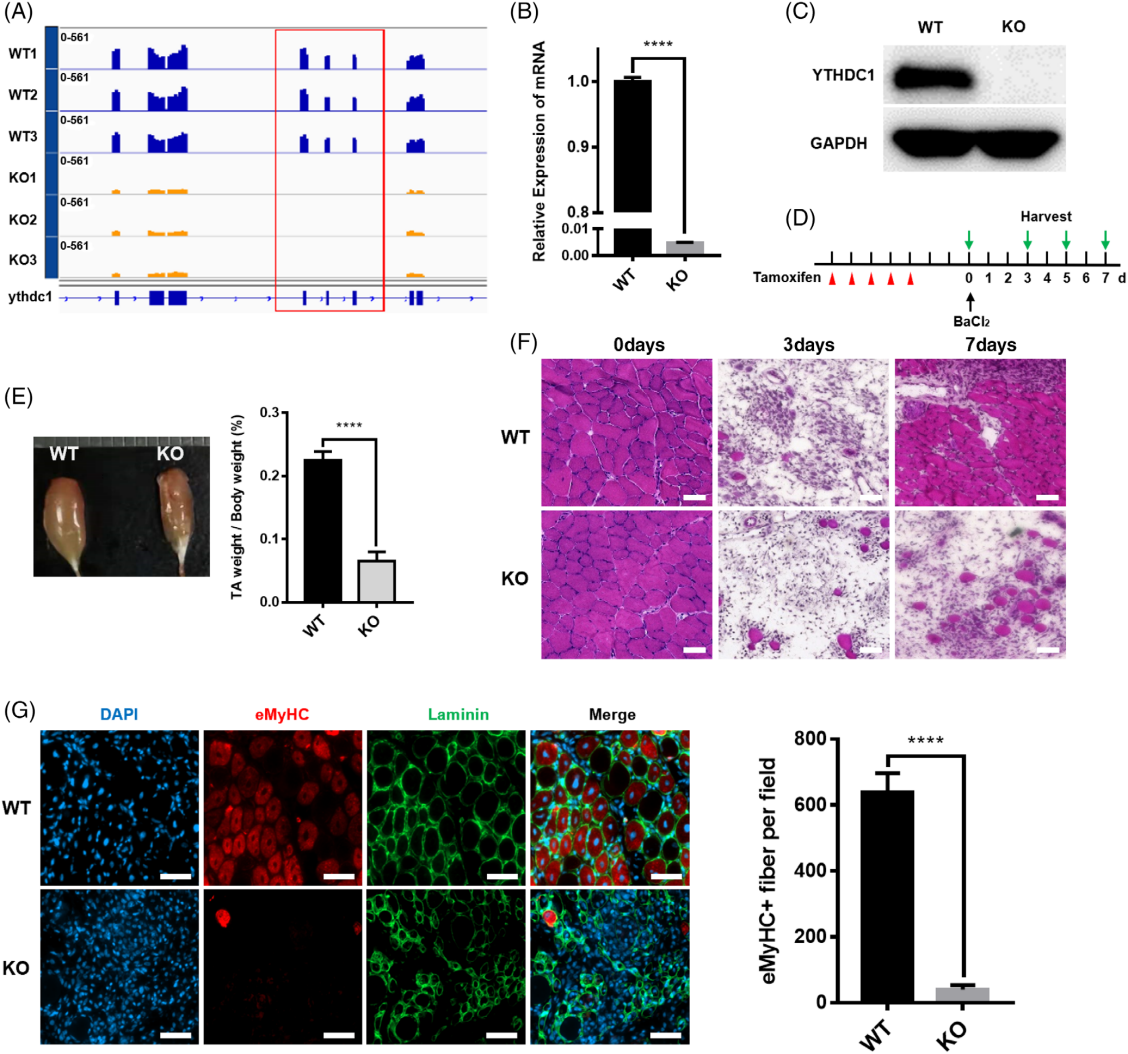
Mice with satellite cell‐specific deletion of Ythdc1 are unable to regenerate skeletal muscles normally. (A) Expression of *Ythdc1* exons in SCs from wild type (WT) and knockout (KO) mice based on RNA‐seq data (WT, *n* = 3; KO, *n* = 3). (B) Quantitative real‐time PCR was performed to detect deleted exons of *Ythdc1* (*n* = 3). (C) Immunoblotting analysis of Ythdc1 protein expression in quiescent SCs isolated from two WT mice or two KO mice pooled together for each group. (D) Schematic outline of tamoxifen administration to obtain WT and KO mice and BaCl_2_‐induced injury. (E) Representative image of regenerating tibialis anterior (TA) muscles 7 days after injury (left panel). Quantification of the weight of above TA muscle (right panel; WT, *n* = 6; KO, *n* = 7). (F) Representative haematoxylin and eosin staining of regenerating TA muscle cross sections 0, 3 and 7 days after injury (WT, *n* = 5; KO, *n* = 5). Scale bar: 50 μm. (G) Immunofluorescence staining of regenerating TA muscle cross sections for embryonic myosin heavy chain (eMHC; red) and laminin (green) by Day 5 post‐injury. The nuclei were counterstained by DAPI (left panel). Quantification of eMHC^+^ myofibers of multiple TA sections (right panel. WT, *n* = 5; KO, *n* = 5). Scale bar: 50 μm. Data represent mean ± SD. Statistical analysis was performed using unpaired two‐tailed Student's *t*‐test (****p < 0.0001).

To investigate the role of Ythdc1 in SCs in adult mice, we developed SCs‐specific and tamoxifen (TMX)‐inducible Ythdc1 conditional KO mice (i.e., *Ythdc1* ^Flox/Flox^: Pax7CreERT2, also abbreviated as Ythdc1 ko or KO thereafter, Figure S1A). Ythdc1 was nearly undetectable in both mRNA and protein level in SCs in KO mice (Figure 1B,C). RNA sequencing (RNA‐seq) data also confirmed that Exons 5–7 of *Ythdc1* was depleted (Figure 1A).

**FIGURE 2:**
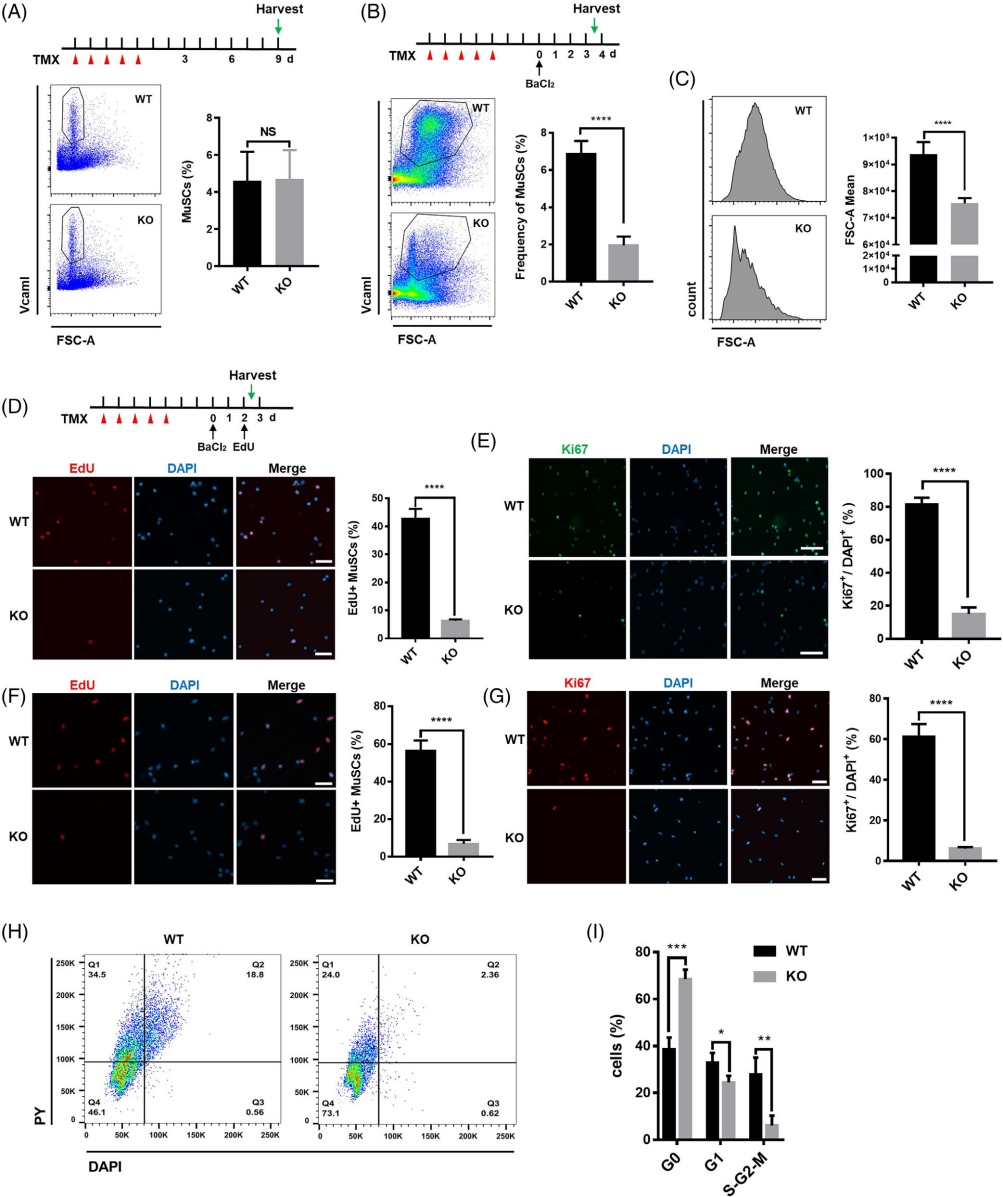
Ythdc1‐null satellite cells (SCs) are unable to exit from quiescence and activate proliferation in vivo and in vitro. (A) Schematic outline of the tamoxifen (TMX) administration to obtain wild type (WT) and knockout (KO) mice in steady state (top panel). Representative flow cytometry results and quantification of SCs in hind limb muscle by Day 9 after TMX administration (bottom panel. WT, *n* = 6; KO, *n* = 6). (B) Schematic outline of the TMX administration to obtain WT and KO mice and BaCl_2_‐induced injury (top panel). Representative flow cytometry results and quantification of SCs in injured hind limb muscle by Day 3.5 post‐injury (bottom panel. WT, *n* = 6; KO, *n* = 6). (C) Representative histograms showing the mean forward scatter (FSC)‐A values of SCs isolated from injured hind limb muscle by Day 3.5 after injury (left panel). Quantification of the mean FSC‐A values (right panel. WT, *n* = 6; KO, *n* = 6). (D) Intraperitoneal injection of 5‐ethynyl‐2′‐deoxyuridine (EdU) at 48 h post‐injury. Then SCs were sorted by fluorescence‐activated cell sorting (FACS), followed by immunofluorescence (IF) staining for EdU (red). The nuclei were counterstained by DAPI (left panel). Quantification of the EdU^+^ SCs by counting ~300 SCs/mouse (right panel. WT, *n* = 5; KO, *n* = 5). Scale bar: 50 μm. (E) IF staining of Ki67 (green) in freshly isolated SCs (2.5 days after injury). The nuclei were counterstained by DAPI (left panel). Quantification of the Ki67 positive SCs by counting ~300 SCs/mouse (right panel. WT, *n* = 5; KO, *n* = 5). Scale bar: 50 μm. (F) SCs were labelled by EdU for 10 h, followed by IF staining for EdU (red). The nuclei were counterstained by DAPI (left panel). Quantification of the EdU^+^ SCs by counting ~400 SCs/mouse (right panel. WT, n = 5; KO, n = 5). Scale bar: 50 μm. (G) SCs were cultured for 46 h, followed by IF staining for Ki67 (red). The nuclei were counterstained by DAPI (left panel). Quantification of the Ki67^+^ SCs by counting ~400 SCs/mouse (right panel. WT, *n* = 5; KO, *n* = 5). Scale bar: 50 μm. (H,I) Cultured SCs were stained with Hoechst 33342 and Pyronin Y and analysed by flow cytometry to determine the cell cycle (WT, *n* = 3; KO, *n* = 3). Data represent mean ± SD. Statistical analysis was performed using unpaired two‐tailed Student's *t*‐test (NS, not significant, *p < 0.05, **p < 0.01, ***p < 0.001, ****p < 0.0001).

First, we determined the number of SCs in homeostasis by fluorescence‐activated cell sorting (FACS). The frequency of SCs in hind limb muscle showed no difference between wild type (WT) and KO mice in homeostasis by Days 9 and 30 post‐five consecutive daily injections of TMX (Figures 2A and S1B). Intriguingly, the number of SCs in mouse hind limb muscle in homeostasis 12 months after TMX administration reduced significantly in the absence of Ythdc1 (Figure S3F,G). While no significant differences were observed in body weight, tibialis anterior (TA) muscle weight, fibre size and total fibre number between WT and KO mice 12 months after TMX administration (Figures S3A–E).

To further assess the function of Ythdc1 in SCs in vivo, acute muscle injury‐regeneration model was employed (Figure 1D). At Days 7 and 28 post‐injury, the regenerated TA muscle was smaller, and mass of regenerated TA decreased in KO mice compared with WT littermates (Figures 1E and S4A,B). By Day 3 post‐injury, haematoxylin and eosin (H&E) staining of TA muscles cross sections showed similar extent of muscle damage and infiltration of inflammatory (Figure 1F). By Days 7 and 28, in KO mice, much fewer regenerating myofibers were observed than that in WT mice (Figures 1F and S4C). Consistently, immunofluorescence (IF) staining of eMHC (embryonic myosin heavy chain), which is expressed in newborn myofibers, manifested that eMHC^+^ regenerating myofibers were rarely seen in KO mice 5 days after injury (Figure 1G). Taken together, our data demonstrate that deletion of Ythdc1 in SCs leads to severe impairment of skeletal muscle regeneration in vivo.

### 2.2 Ythdc1 is required for the proliferation of SCs in vivo and in vitro

To explore the reason of regeneration failure in KO mice, we quantified the number of SCs in injured mice muscle by FACS. Three and a half days after injury, we observed dramatic decrease in frequency of SCs in injured hind limb muscle in Ythdc1 KO mice (Figure 2B). And the expression of genes related to myogenesis in Ythdc1‐null SCs, such as *MyoG*, *Tnni2*, *Acta1* and *Myh3*, was much lower than that in WT SCs after injury (Figure S1C).

As we know, in response to acute muscle damage, the quiescent SCs exit dormancy, undergo proliferation, and then differentiate and fuse to form newborn multinucleated myotubes.^4^ Thus, at first, we examined 5‐ethynyl‐2′‐deoxyuridine (EdU) incorporation in vivo upon injury. Nearly, 40% of SCs isolated from injured WT mice were labelled by EdU, whereas only about 5% of Ythdc1‐null SCs were labelled (Figure 2D). Ki67 staining of SCs from injured mice confirmed cell cycle arrest in Ythdc1‐null SCs, more than 80% of SCs in injured WT mice expressed Ki67 protein highly, <20% of SCs from KO mice did so (Figure 2E). Furthermore, the size of SCs isolated from WT mice was much bigger than that isolated from KO mice upon injury (Figure 2C).

In addition, SCs were isolated freshly from uninjured mice by FACS at Day 9 post‐TMX treatment, and cultured in growth medium to determine EdU incorporation and ki67 expression in vitro. First, we confirmed that almost 97.1% cells sorted by FACS are Pax7‐positive SCs (Figure S1D). The results showed that the number of EdU^+^ cells in Ythdc1‐null SCs decreased remarkably (WT [55.4%] vs. KO [6.8%]; Figure 2F). When stained for Ki67 in cultured SCs, the abundance of Ki67^+^ SCs were declined dramatically in Ythdc1‐null SCs (WT [58.9%] vs. KO [6.5%]; Figure 2G). By Hoechst 33342 and Pyronin Y staining for DNA/RNA content, the results showed that much more cultured SCs isolated from KO mice were blocked in G0 state with low RNA content compared with that from WT mice (Figure 2H,I). Taken together, our data above indicate that Ythdc1 is indispensable for proliferation of SCs in vivo and in vitro.

### 2.3 Ythdc1 regulates splicing and promotes intron retention in SCs

**FIGURE 3:**
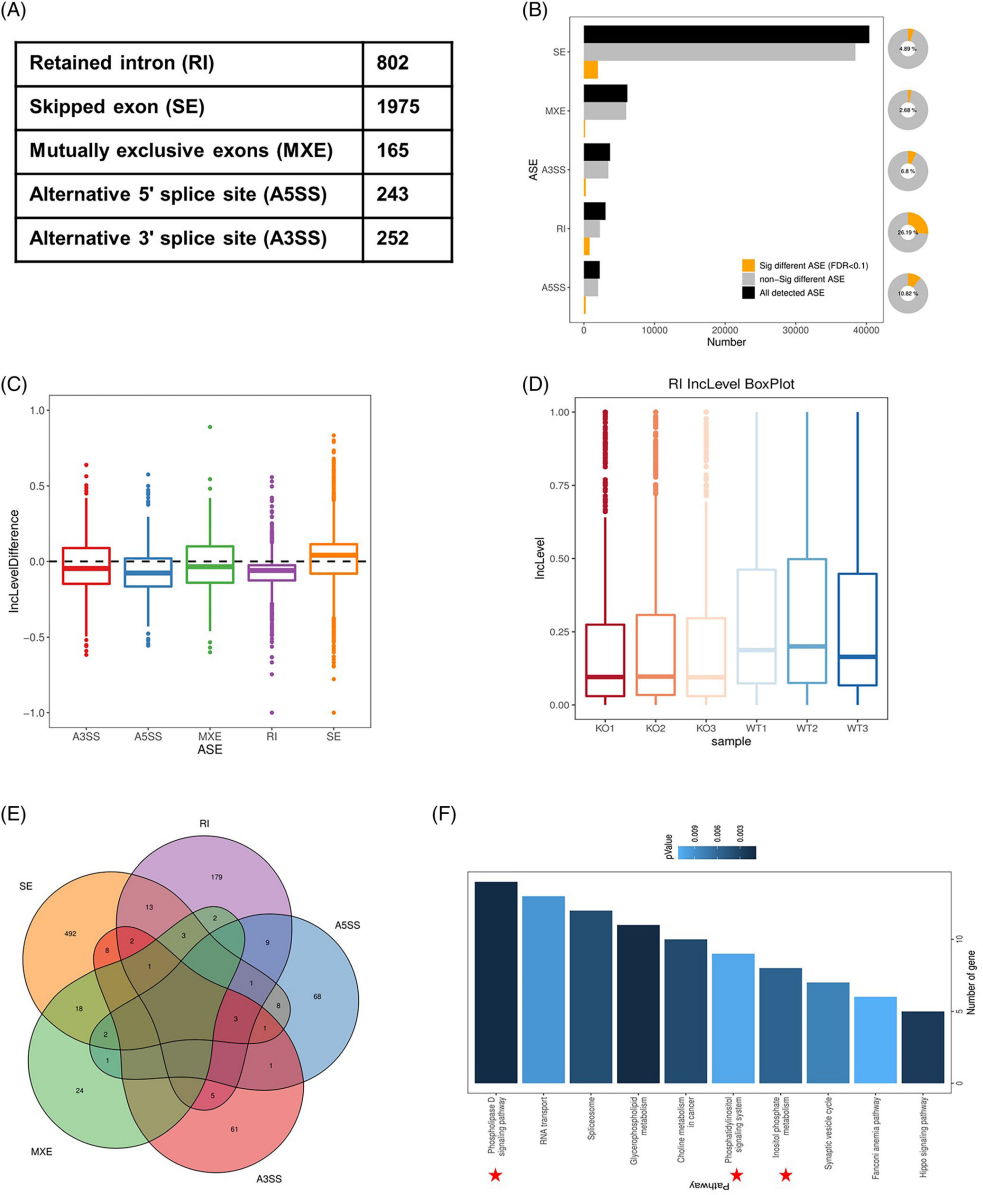
Ythdc1 regulates mRNA alternative splicing and promotes intron retention in satellite cells (SCs). (A,B) Summary of significantly differential alternative splicing events (ASEs) in SCs isolated from wild type and knockout mice identified by replicate multivariate analysis of transcript splicing. (C) Difference of inclusion level of five‐type ASEs (Inclusion level of ASE in Ythdc1‐null SCs—Inclusion level of ASE in SCs). (D) Box plot of inclusion level of intron in SCs (WT, *n* = 3; KO, *n* = 3). (E) Venn diagram of genes with high‐confidence differential ASEs. (F) KEGG pathways analysis of genes with high‐confidence differential ASEs.

To dissect the underlying mechanism, RNA‐seq was performed with RNAs extracted from SCs isolated from uninjured mice. Given that the key role of Ythdc1 is to regulate alternative splicing of its target mRNA,^32^ we applied rMATS (replicate multivariate analysis of transcript splicing) to detect differential alternative splicing in the absence of Ythdc1 in SCs.^33^ We identified a total of 3437 significantly differential alternative splicing events (ASEs) including SE, RI, MXE, A5SS and A3SS (Figure 3A,B). We found that 26.19% of detected retained intron events showed significant differences in SCs between WT mice and KO mice (Figure 3B). And in Ythdc1‐null SCs, inclusion level of intron decreased distinctly, whereas other types of ASE did not (Figures 3C,D and S2A–D). In total, differential ASEs with high confidence located in 844 genes (Figure 3E).

**FIGURE 4:**
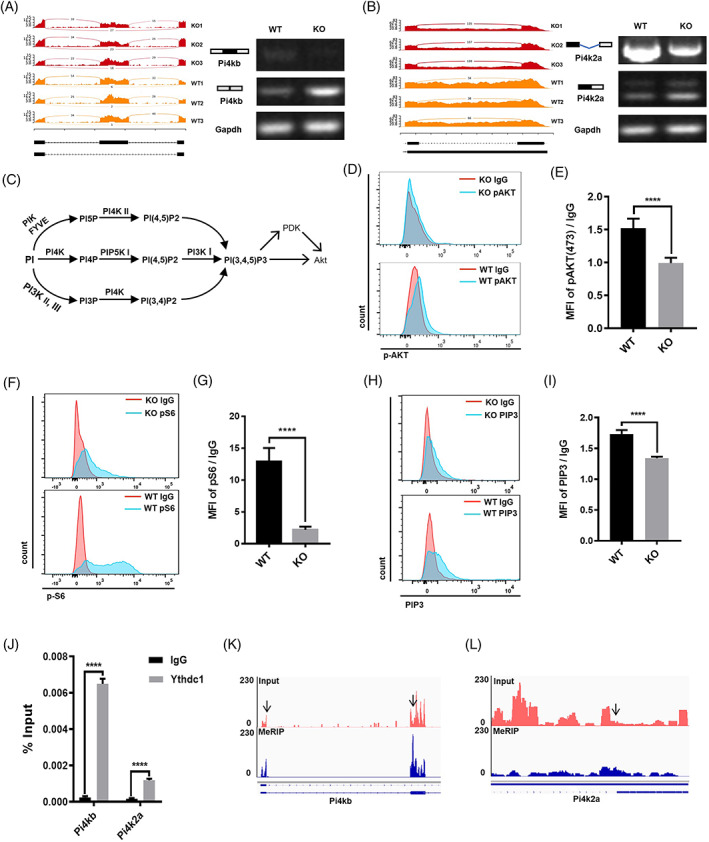
Ythdc1‐null satellite cells (SCs) show deficiencies in PI4K–Akt–mTOR signalling. (A,B) Gene track view and reverse‐transcription (RT)‐PCR validation of exon skipping in *Pi4kb* (A) and intron retention in *Pi4k2a* (B). (C) Phosphatidylinositol metabolism and Akt. (D) Phosphorylated Akt‐473 in SCs by Day 2.5 post‐injury were determined by fluorescence‐activated cell sorting (FACS). (E) Quantification of phosphorylated Akt‐473 showed in (D; wild type [WT], *n* = 5; knockout [KO], *n* = 5). (F) pS6 protein in SCs by Day 2.5 post‐injury were determined by FACS. (G) Quantification of pS6 protein showed in (F; WT, *n* = 5; O, *n* = 5). (H) PI 3,4,5‐trisphosphate (PIP3) content in SCs by Day 30 post‐TMX administration was determined by FACS. (I) Quantification of PIP3 content showed in (H; WT, *n* = 4; KO, *n* = 4). (J) RNA immunoprecipitation‐RT‐qPCR assay with antibody against Ythdc1 was performed in C2C12 myoblast (*n* = 3). (K,L) IGV tracks displaying N6‐methyladenosine‐enriched *Pi4kb* (K) and *Pi4k2a* (L) in C2C12 myoblast. Data represent mean ± SD. Statistical analysis was performed using unpaired two‐tailed Student's *t*‐test (****p < 0.0001).

By KEGG analysis of genes with high‐confidence ASEs, we paid attention to PI signalling (Figure 3F). A lot of genes involved in PI metabolism showed different inclusion level of ASE. Among of those genes, inclusion level of exon of *Pi4kb* decreased remarkably in Ythdc1‐null SCs, so did intron of *Pi4k2a* (Figure 4A,B). Then, we performed RT‐PCR to validate rMATS‐identified ASEs of *Pi4kb* and *Pi4k2a*. RT‐PCR analysis confirmed the alteration of splicing in Ythdc1‐null SCs (Figure 4A,B). The second exon of *Pi4kb* was mostly skipped in Ythdc1‐null SCs but only a little in WT (Figure 4A). Intron between Exons 9 and 10 of *Pi4k2a* preferred to retain in WT SCs (Figure 4B). Taken together, Ythdc1 regulates mRNA splicing and promotes intron retention in SCs.

### 2.4 Akt–mTOR pathway is interfered in Ythdc1‐null SCs

PI lipids have been shown to be crucial signal molecules in response to extracellular growth factor. PI and its derivatives involve diverse cellular processes including cell proliferation, cell differentiation, cell death, membrane trafficking, cell‐matrix adhesion and gene expression.^34^, ^35^ PI 4‐kinase (PI4K) played a critical role in PI metabolism which had an effect on the production of PIP3 (Figure 4C).^36^, ^37^ Our data showed that the transcription initiation site in the second exon of *Pi4kb* was skipped, which may give rise to loss of function of Pi4kb. Intron retention of *Pi4k2a* altered 3′ untranslated region, which may regulate Pi4k2a feature. Thus, PIP3 content in Ythdc1‐null SCs may be lower than that in normal SCs. Without enough PIP3, activation of Akt–mTOR would be disturbed, which leads to failure in quiescence exit in SCs.^11^, ^38^, ^39^, ^40^

To test whether Akt–mTOR was interfered, we determined phosphorylation of Akt in SCs by FACS at Day 2 post‐BaCl_2_‐induced muscle injury in vivo. The results showed that activated Akt in Ythdc1‐null SCs, phosphorylated Akt on serine 473 (pAkt‐473), was less than that in control cells (Figure 4D,E). In cultured SCs isolated from mice, pAkt‐473 was also downregulated in the absence of Ythdc1 in SCs (Figure S2G). Next, we quantified phosphorylated ribosomal S6 protein (p‐S6), a direct target of Akt–mTOR pathway, by FACS at same time point. The protein level of pS6 of Ythdc1‐null SCs was distinctly lower than that in control cells in vivo (Figure 4F,G). Consistently, immunofluorescence staining and western blot analysis of p‐S6 in cultured SCs for 36 h in vitro confirmed interference of Akt–mTOR pathway (Figure S2E,F). Taken together, activation of Akt–mTOR pathway in Ythdc1‐null SCs is disturbed, which is responsible for failure in quiescence exit.

To verify the hypothesis that depletion of Ythdc1 in SCs decreased the production of PIP3 via regulation of Pi4kb and Pi4k2a directly, at first, we determined the abundance of PIP3 in SCs via immunofluorescence staining. The results revealed that PIP3 content in Ythdc1‐null SCs was significantly reduced compared to that in control cells (Figure 4H,I). Next, we performed RNA immunoprecipitation (RIP)‐RT‐qPCR assay with antibody against Ythdc1 to examine the interaction between Ythdc1 protein and target mRNAs. The results validated that Ythdc1 markedly enriched *Pi4kb* and *Pi4k2a* mRNA compared with the immunoglobulin G (IgG) pull‐down control in C2C12 myoblast (Figure 4J), demonstrating that Ythdc1 interacted with *Pi4kb* and *Pi4k2a* mRNA. Furthermore, we performed m6A methylation peak calling using algorithm exomePeak with MeRIP‐seq data from previous study.^23^, ^41^ We uncovered that m6A modification of *Pi4kb* and *Pi4k2a* enriched in the exon, nearby which ASEs could be found (Figure 4K,L). In addition, overexpression of WT Ythdc1, not the YTH‐domain‐mutants which impede recognition and binding of m6A modified RNA and Ythdc1, rescued the proliferation deficiency of Ythdc1‐null SCs, which implicated that Ythdc1 regulates the function of SCs in a m6A‐dependent manner (Figures S5A,B). Taken together, we demonstrate that Ythdc1 binds to m6A‐modified *Pi4kb* and *Pi4k2a* to regulate production of PIP3, which leads to failure in activation of Akt–mTOR pathway in Ythdc1‐null SCs.

## 3 DISCUSSION

In this study, we used conditional KO mice to uncover that Ythdc1, one of the readers of RNA m6A modifications, is required for SC proliferation to repair damaged skeletal muscle tissues. Our results are consistent with conclusions from previously published reports,^24^, ^25^ which showed that the m6A writer Mettl3 regulates the proliferation of muscle stem cells during mouse muscle regeneration, and that the m6A eraser Fto controls mouse muscle development. Although previous studies implicated m6A modification in myogenesis, we did not observe obvious developmental defects in SC‐specific Ythdf1‐null mice. Our previous study implicated that loss of Ythdf2 enhances regenerative capacity of adult HSC,^42^, ^43^ but showed no difference in muscle regeneration. It is also unclear exactly how Fto regulates the mTOR pathway in myogenesis.^25^ Our results suggest at least one possible mechanism is that m6A‐modified Pi4k2a and Pi4kb mRNAs regulate PI4K‐Akt–mTOR signalling. These results indicate that a sophisticated system exists to mediate m6A modifications' effects on biological processes. More studies will be needed to dissect the role of writers, erasers and readers of m6A modifications in different contexts, including skeletal muscle development and regeneration.

Ythdc1 is a reader protein of m6A modification which is localized in the nucleus. It is involved in mRNA transcription, splicing, nuclear export, polyadenylation and degradation.^27^, ^28^, ^32^ In this study, we found that Ythdc1 promotes intron retention in SCs in general. Specifically, for Pi4k2a and Pi4kb, Ythdc1 binds directly to their m6A‐modified mRNAs to regulate their alternative splicing. PI4K is a key regulator of PI metabolism,^36^ and indeed we observed that PIP3 levels are deficient in Ythdc1‐null SCs. It is known that PIP3‐associated metabolites play a central role in signal transduction in cellular processes.^44^ Importantly, disorder of PIP3 metabolism correlates with a variety of human pathologies including neurodegeneration, primary immunodeficiencies and cancer.^35^, ^45^ Thus, it is possible that Ythdc1 regulation of PI4K alternative splicing and PIP3 metabolism could play important roles in other contexts as well, and serve as a potential therapeutic target for PIP3‐related diseases.

In summary, we discovered that Ythdc1 is indispensable for mouse muscle regeneration. Upon loss of Ythdc1, SCs are unable to exit from quiescence. Ythdc1 binds to m6A‐modified Pi4k2a and Pi4kb mRNA to regulate their alternative splicing and thus PI4K activity and PIP3 metabolism. Depletion of Ythdc1 in SCs results in decreased production of PIP3. PIP3, a critical second messenger, triggers Akt phosphorylation. Therefore, Ythdc1 regulates SCs' exit from quiescence to proliferate during muscle regeneration, by modulating PI4K alternative splicing, PIP3 levels, and Akt–mTOR signalling. Our findings provide a new perspective on how nutrient‐responsive RNA methylation can regulate Akt–mTOR signalling during stem cell proliferation and adult tissue regeneration.

## 4 EXPERIMENTAL MODEL AND SUBJECT DETAILS

### 4.1 Animals

*Ythdc1* ^flox/flox^ mice were a kindly gift from Professor Bin Shen (Nanjing Medical University). Pax7^tm1(cre/ERT2)Gaka^ mice were kindly provided by Professor Hongbo Zhang (Sun Yat‐sen University). *Ythdc1* ^flox/flox^: Pax7CreERT2 mice were generated by crossing *Ythdc1* ^*f*lox/flox^ mice with Pax7^tm1(cre/ERT2)Gaka^ mice. Mice were genotyped with primers shown in Table S1. In order to delete Ythdc1 specifically in SC, experimental mice were administered intraperitoneally with 2 mg TMX (Sigma) dissolved in corn oil per 20 g body weight for 5 consecutive days. All mice were housed in specific pathogen‐free animal facility. Procedures and protocols involving mice were approved by the Animal Care and Ethics Committee of Jinan University. If not stated specifically, 2–4 months old and gender‐matched mice were used for all experiments.

### 4.2 Cell culture

Mouse C2C12 myoblast cell lines were cultured in dulbecco’s modified eagle’s medium (DMEM) medium with 10% fetal bovine serum, 1% penicillin/streptomycin (Gibco) at 37°C, 5% CO_2_. Primary myoblast cells were cultured in Ham's F10 Nutrient Mixture (Sigma) supplemented with 20% fetal bovine serum and 5 ng/mL bFGF (Promega; growth medium) at 37°C, 5% CO_2_. Petri dishes for primary myoblast cells culture were pre‐coated with extracellular matrix (ECM; Sigma) gel at 4°C.

## 5 METHOD DETAILS

### 5.1 Muscle injury and regeneration model

Adult mice were anaesthetized with isoflurane during surgery. Muscle injury and regeneration was induced by intramuscular injection of 50 μL of 1.2% (wt/vol) BaCl_2_ solution into TA muscles. After injury, TA muscles were harvested at various time points for further analysis.

### 5.2 Isolation of adult muscle SCs by FACS

Adult muscle SCs were isolated as previously described.^46^ Briefly, Hind limb muscles dissected from mice were gently minced, digested by collagenase II (800 U/mL) in washing medium (Ham's F10 with 10% horse serum) for 90 min in a shaking water bath at 37°C. Then, digested muscles tissue was triturated and washed in the washing medium followed by further digestion with collagenase II (100 U/mL) and dispase (1 U/mL) for 30 min. Digested tissue was then passed through a 20‐Gauge needle for 15 times and filtered through a 40 μm cell strainer. Single‐cell suspension was stained by antibody cocktail (FITC anti‐CD31, FITC anti‐CD45, PE/Cy7 anti‐Sca1 and biotin anti‐Vcam1). The Vcam1 signal was amplified with PE streptavidin. Red blood cells were lysed by RBC lysis buffer. Satellite cells (Sca1−/CD31−/CD45−/Vcam1+) were purified by FACS sorting with BD FACS Arial III.

### 5.3 Quantitative real‐time PCR

Total RNA was extracted from SCs using the RNAqueous®‐Micro Kit (Life Technology). And then reverse transcription (RT) was performed using PrimeScript™ RT Master Mix (TaKaRa). The qPCR analysis was performed with SsoFast™ EvaGreen® Supermix (Bio‐Rad) on QuantStudio 6 Flex system (Applied Biosystems). GAPDH mRNA was used for normalization. Primers used for real‐time qPCR are shown in Table S1.

### 5.4 Western blot

Cells were lysed in RIPA buffer with protease inhibitor and phosphatase inhibitor. The protein samples were separated by sodium dodecyl sulfate (SDS)‐polyacrylamide gel electrophoresis (PAGE) gels, transferred onto polyvinylidene fluoride (PVDF) membranes (Bio‐Rad), and blocked with TBST containing 5% milk for 1 h at RT, then probed with primary antibodies (Key Resources Table). The membranes were then incubated with secondary antibodies (Key Resources Table) for 1 h at RT, and detected on an Amersham Imager 600 System (GE).

### 5.5 H&E staining and immunofluorescence staining

TA muscle tissues from WT or KO mice were isolated and dehydrated in 30% (wt/vol) sucrose solution. Then, the dehydrated TA muscles were frozen in optimal cutting temperature (OCT) compound. Cross sections (10 μm) of frozen TA muscle were used for H&E staining and immunofluorescence staining. For H&E staining, the slides were first stained in haematoxylin for 8 min, washed in running tap water for 5 min and then counterstained in eosin‐phloxine solution for 1 min. The specimens were dehydrated in graded ethanol (70%–100%) and Xylene, and then covered with mounting medium. For immunofluorescence staining of cryo‐sections, immunofluorescence staining was performed as described in the article.^47^ Immunofluorescence staining of Ki67 and Pax7, cells were fixed with 4% paraformaldehyde (PFA) for 15 min at RT. Then cells were permeabilized with 0.5% Triton X‐100 in phosphate buffer solution (PBS) for 20 min, blocked with 3% IgG‐free bovine serum albumin (BSA) and 0.1% Triton X‐100 in PBS for 1 hour (blocking solution) at RT, followed by incubation with antibody against Ki67 (Abcam) or Pax7 (DSHB) at 4°C overnight. After washing with PBS, the samples were incubated with secondary antibodies (Key Resources Table) for 1 h at RT. Immunofluorescence staining of pS6 (CST) was performed according to the manufacturer's instructions. The nuclei were counterstained with DAPI.

### 5.6 EdU assay in vivo and in vitro

In vivo, by Day 2 post‐injury, mice were injected intraperitoneally with EdU (Beyotime, 0.05 mg/g body weight), followed by FACS isolation of SCs 12 h later. EdU was detected with the Click‐iT EdU imaging kit (Invitrogen). In vitro, an equal number of SCs sorted by FACS isolated from WT or KO mice were cultured in growth medium for 36 h and labelled with EdU for 10 h. Then EdU assay was performed with the Click‐iT EdU imaging kit according to the manufacturer's instructions.

### 5.7 Pyronin Y and Hoechst 33342 staining

Satellite cells sorted by FACS were cultured for 46 h in growth medium. Then cells were detached by Trypsin, resuspended in Pyronin Y staining medium (Ham's F10 + 20% FBS + 20 mM HEPES + 50 μM verapamil). The samples were incubated with 10 μg/mL of Hoechst 33342 for 1 h at 37°C. Then Pyronin Y was directly added to the samples (final concentration is 10 μg/mL) for 1 h at 37°C followed by FACS analysis.

### 5.8 Validation of ASEs

For validation of ASEs, RT‐PCR were conducted. The PCR cycles varied for each ASE because of the different abundance of transcript. Primers used for RT PCR are shown in Table S1.

### 5.9 Analysis of protein or PIP3 level by flow cytometry

Satellite cells were isolated as described above. FITC anti‐Sca1 instead of PE/Cy7 anti‐Sca1 and APC‐Cy7 streptavidin instead of PE streptavidin were used for the staining. Then immunofluorescence staining of intracellular phosphor‐Akt (Ser473; CST) and pS6 (CST) for flow cytometric analysis were performed according to the manufacture's protocol. Immunofluorescence staining for PIP3, cells stained with above antibodies were fixed in 4% formaldehyde for 20 min at RT, washed three times with PBS, and then permeabilized with 0.2% Triton X‐100 in PBS for 10 min at RT. Samples were blocked with 3% IgG‐free BSA and 0.1% Triton X‐100 in PBS for 1 h at RT, followed by incubation with antibody against PIP3 (Echelon Biosciences) or anti mouse IgG isotype control (Santa Cruz) at RT for 90 min. After washing with PBS, the samples were incubated with secondary antibodies (Key Resources Table) for 30 min at RT followed by FACS analysis.

### 5.10 RIP real‐time qPCR

C2C12 myoblast cultured in DMEM with 10% FBS, 1% penicillin/streptomycin were collected for further study. All the specific manipulations were performed according to the protocol of Imprint RIP Kit (RIP‐12RXN, Sigma‐Aldrich). The samples were incubated with antibody against Ythdc1 (CST) or anti Rabbit IgG isotype control (CST). The primers for RIP real‐time qPCR were listed in Table S1.

### 5.11 Analysis of m6A methylation sites by exomePeak algorithm

First, we downloaded the online raw data from DNA Data Bank of Japan database (accession no: DRA005057). Raw sequencing reads were mapped to reference mouse genome (mm10) using Hisat2 software. PCR duplication and low‐quality reads were removed by using SAMtools software. Mapped and filtered reads were subjected to the methylation peak caller exomePeak to predict m6A methylation sites.

### 5.12 RNA‐seq and data analysis

Adult *Ythdc1* ^flox/flox^ mice and their *Ythdc1* ^flox/flox^: Pax7CreERT2 littermates were treated with TMX for 5 consecutive days. Nine days after the last TMX injection, SCs were isolated by FACS as described above. RNA extraction and sequencing libraries were prepared as described in Zhencan Shi et al.^48^ Sequencing was performed on NovaSeq platform (pair‐end with 150 bp). Sequencing reads were mapped to the mouse genome assembly (mm10) using Hisat2 software. Replicate multivariate analysis of transcript splicing (rMATS) was applied to RNA‐seq data to detect differential ASEs. ASEs (false discovery rate (FDR) <0.05) with | IncLevelDiff | (KO–WT) > 0.1 were considered significant difference. Sequencing data of the experiment are deposited at NIH's Sequence Read Archive and have been assigned a BioProject accession number PRJNA776399.

## 6 QUANTIFICATION AND STATISTICAL ANALYSIS

All data represent mean ± SD. Statistical analysis was performed using unpaired two‐tailed Student's *t*‐test. For all tests, *p*‐values < 0.05 were considered significant.

## AUTHOR CONTRIBUTIONS

Hu Wang, Jinping Zheng and Zhenyu Ju initiated the study and developed the concept of the article. Jin Liu, Jinping Zheng and Hu Wang designed the experiments. Jin Liu, Hongna Zuo, Ziliu Wang, Wei Wang, Xuezhen Qian and Wanling You performed experiments and acquired data. Hu Wang, Jin Liu, Liquan Hong and Hongna Zuo analysed and interpreted the data. Guanzheng Luo and Yingyuan Xie analysed RNA‐seq data. Di Peng, Yingyuan Xie and Jian Ren analysed m6A methylation sites by exome Peak algorithm. Jin Liu, Hu Wang, Bin Shen, Liquan Hong, Huiling Lou, Jinping Zheng and Zhenyu Ju wrote and revised the article. Zhenyu Ju and Hu Wang supervised the study.

## CONFLICT OF INTEREST STATEMENT

The authors declare no competing interests.

## Supporting information


**Data S1:** Supporting Information.

## Data Availability

Further information and requests for resources and regents should be directed to and will be fulfilled by the Lead Contact, Zhenyu Ju (zhenyuju@163.com).
