# Supplementary material for: The m6A reader YTHDC1 regulates muscle stem cell proliferation via PI4K–Akt–mTOR signalling
Source: Cell Prolif. 2023 Feb 1;56(8):e13410. doi: 10.1111/cpr.13410 (PMC10392063; doi:10.1111/cpr.13410)
Supplement: Supplementary file 1 — Data S1: Supporting Information. [file CPR-56-e13410-s001.docx]

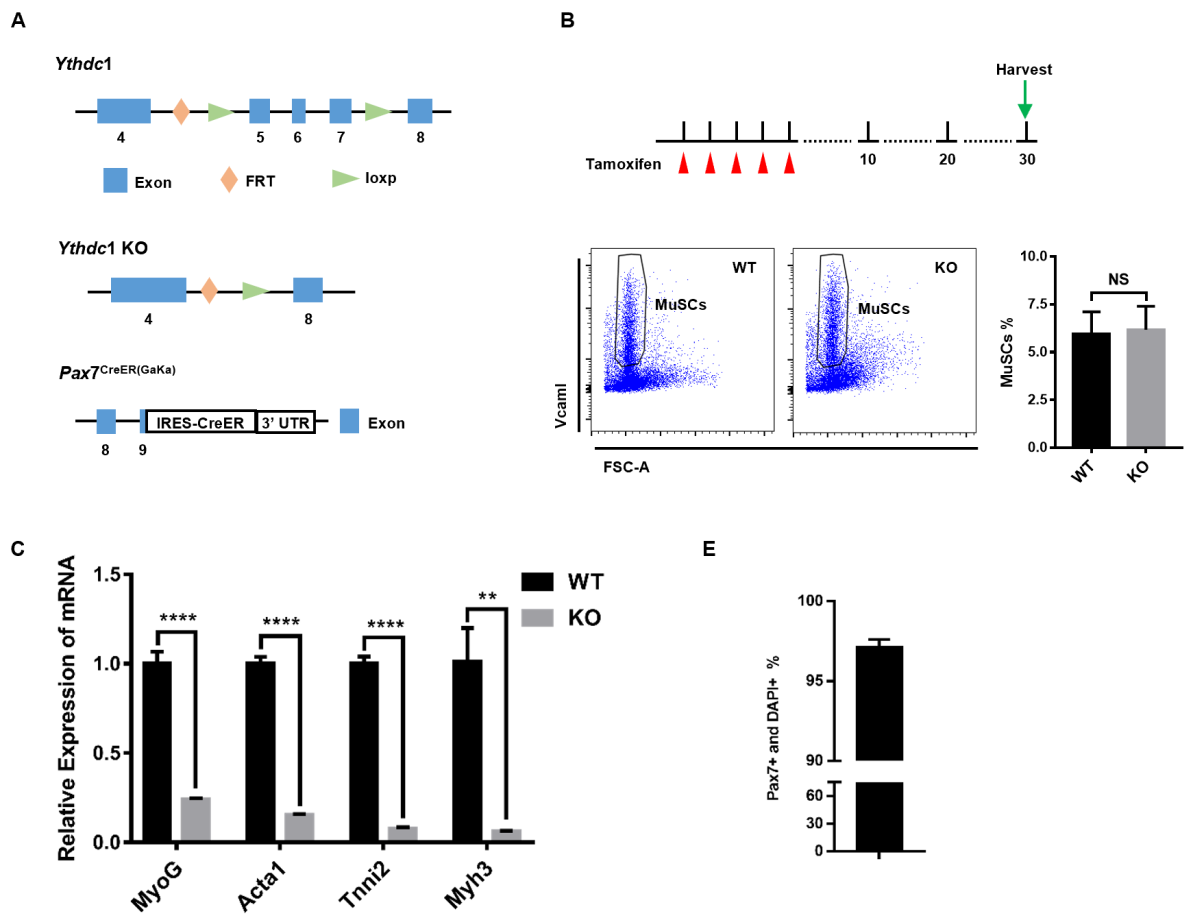


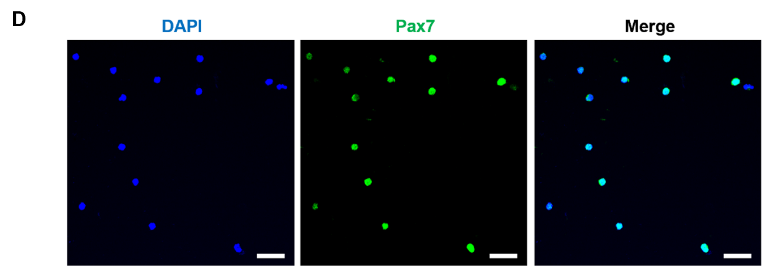


Figure S1. Loss of Ythdc1 in satellite cells impairs muscle regeneration. Related to Figure 1 and Figure 2.

(A) Top: the outline of the floxed exon at the *Ythdc1* locus in non-inducible mice. Middle: the outline of the floxed exon at the *Ythdc1* locus in inducible satellite cells-specific Ythdc1 knockout mice. Bottom: the outline of the location of CreERT2 in Pax7^cre/ERT2(GaKa)^ mice.

(B) Schematic outline of the TMX administration to obtain WT and KO mice in steady state (top panel). Representative flow cytometry results and quantification of satellite cells in hind limb muscle by day 30 after TMX administration (bottom panel. WT, n = 6；KO, n=6).

(C) Quantitative real time PCR to determine the expression of genes related to myogenesis by day 3.5 after injury (WT, n = 3；KO, n=3).

(D) Satellite cells sorted by FACS were cultured for 24 h, followed by immunofluorescence staining for Pax7. Scale bar: 20μm.

(E) Quantification of Pax7 positive cells showed in D by counting ~300 cells/mouse (n = 3).

Data represent mean ± SD. Statistical analysis was performed using unpaired two-tailed Student’s t test (NS: not significant, ∗∗p < 0.01, ∗∗∗∗p < 0.0001).


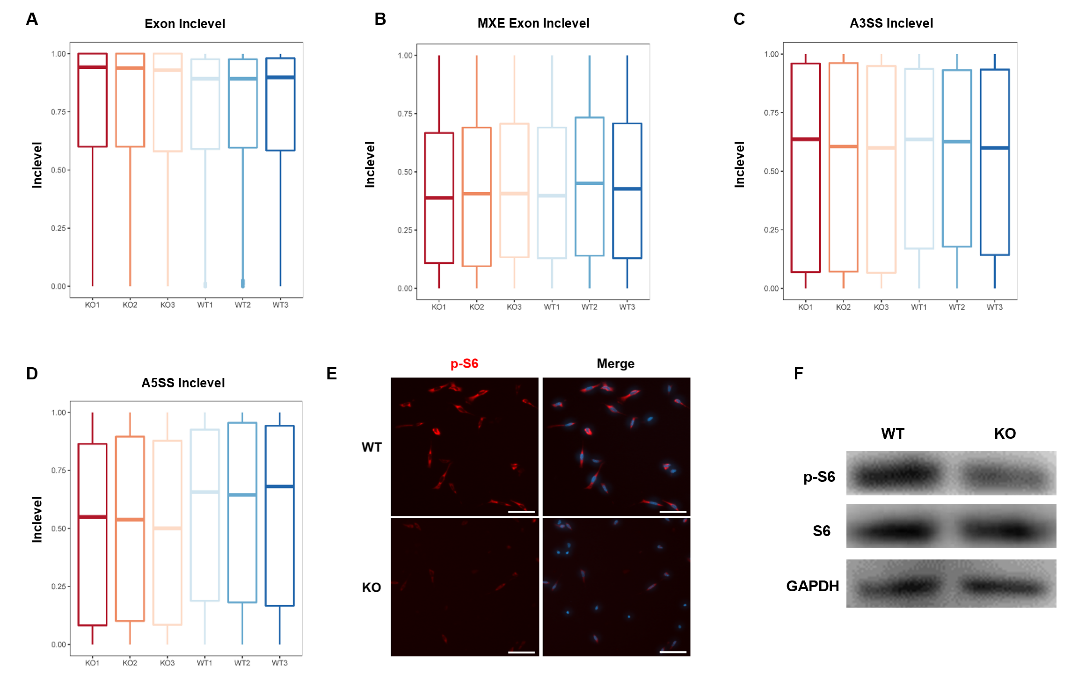


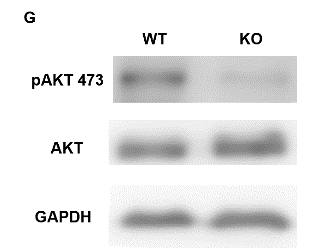


Figure S2. Ythdc1 regulates alternative splicing in satellite cells and mTOR pathway is interfered in Ythdc1-null satellite cells. Related to Figure 3 and Figure 4.

(A-D) Box plot of inclusion level of exon (A), mutually exclusive exons (B), alternative 3’ splice site (C) and alternative 5’ splice site (D) in satellite cells (WT, n = 3；KO, n=3)

(E) Satellite cells sorted by FACS from WT mice or KO mice were cultured for 24 h, followed by immunofluorescence staining for phosphorylated ribosomal S6 protein. Scale bar: 50μm.

(F) Immunoblotting analysis of S6 and pS6 expression in satellite cells. Satellite cells isolated from 2 WT mice or 2 KO mice were cultured for 24h, then lysates extracted from cultured satellite cells were pooled together for each group.

(G) Immunoblotting analysis of Akt and pAkt 473 expression in satellite cells. Satellite cells isolated from 2 WT mice or 2 KO mice were cultured for 24h, then lysates extracted from cultured satellite cells were pooled together for each group


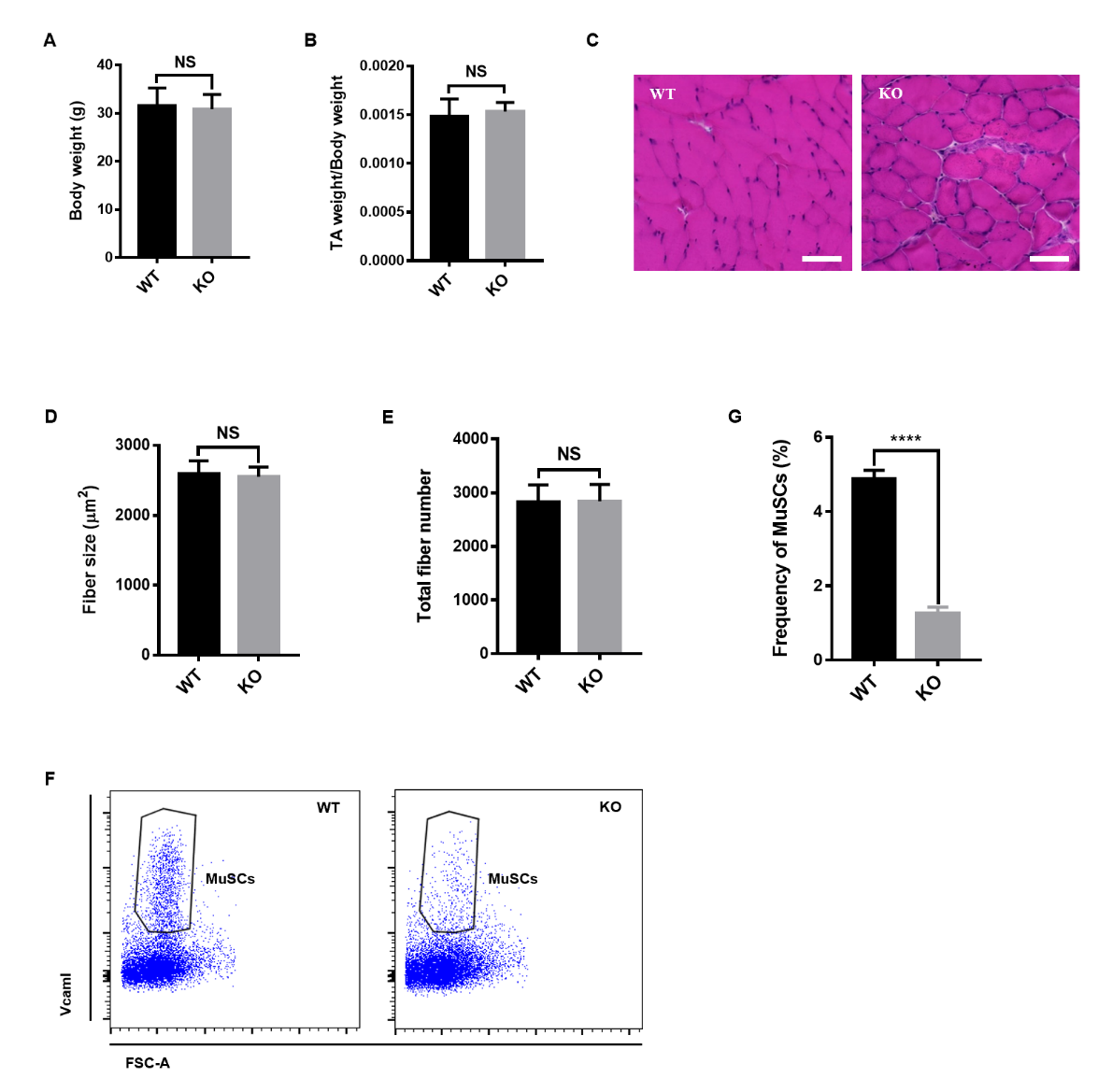


Figure S3. The number of satellite cells decreases 12 months after tamoxifen administration in tamoxifen-inducible Ythdc1 conditional knockout mice

1. Quantification of the body weight of mice 12 months after TMX administration (WT, n = 7；KO, n=6).
2. Quantification of the TA muscle weight of mice 12 months after TMX administration (WT, n = 7；KO, n=6).
3. Representative H&E staining of TA muscle cross-sections of mice 12 months after TMX administration (WT, n = 7；KO, n=6). Scale bar: 50μm.
4. Quantification of myofibre size of mice 12 months after TMX administration, evaluated by the cross-sectional area of TA muscle (WT, n = 7；KO, n=6).
5. The number of myofiber of TA muscle of mice 12 months after TMX administration (WT, n = 7；KO, n=6).

(F and G) Representative flow cytometry results and quantification of MuSCs in hind limb muscle 12 months after TMX administration (WT, n = 3；KO, n=3).


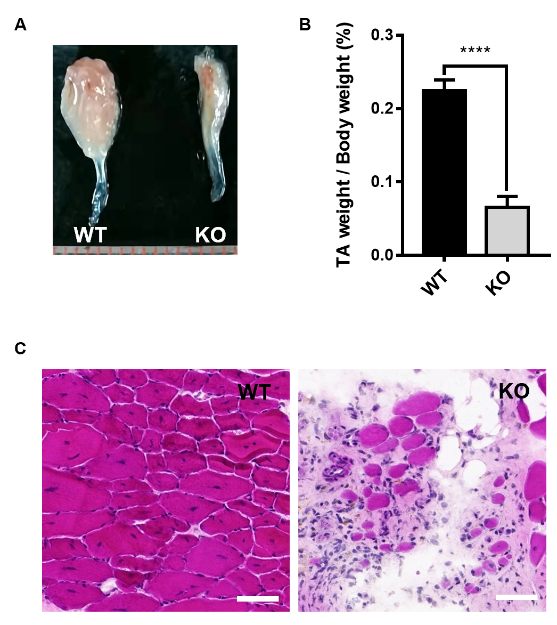


Figure S4. Mice that delete Ythdc1 in satellite cells lose the function to reconstitute damaged muscle tissue. Related to Figure 1.

1. Representative image of regenerating TA muscles 28 days after injury.
2. Quantification of the weight of above TA muscle (WT, n = 5；KO, n=5).
3. Representative H&E staining of regenerating TA muscle cross-sections 28 days after injury (WT, n = 5；KO, n=5). Scale bar: 50μm.


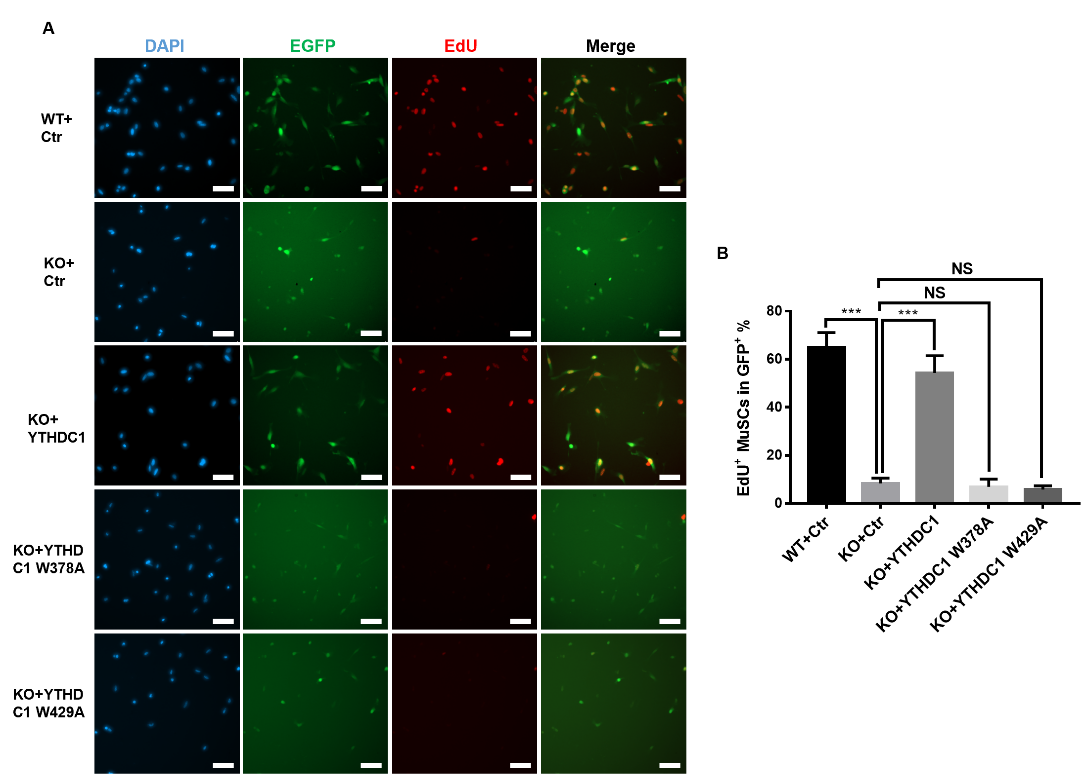


Figure S5. Overexpression of wild-type Ythdc1, not the YTH-domain-mutants, rescued the proliferation deficiency of Ythdc1-null satellite cells

1. Cultured satellite cells were transfected by lentivirus, then labeled by EdU for 10h, followed by immunofluorescence staining for EdU (red). The nuclei were counterstained by DAPI (WT, n =3；KO, n=3). Scale bar: 50μm.
2. Quantification of the EdU^+^ SCs in GFP^+^ cells by counting ~300 SCs/mouse (right panel. WT, n =3；KO, n=3).

**Table S1.** Nucleotide sequence of genotyping primers, real time qPCR primers and RT-PCR primers.

| **Nucleotide sequence of genotyping primers** | |
| --- | --- |
| **Gene** | **Nucleotide sequence (5’-3’)** |
| *Ythdc1*^flox/flox^ | Forward: CATCTCTCCAGCCCGGTAAA |
|  | Reverse: GTGCTACACTAAGTCCTGTGAC |
| Pax7 CreERT2 | Forward: CTGAAGGATGCCCAGAAGGTA |
|  | Reverse: GCCGCATAACCAGTGAAACAG |
| **Nucleotide sequence of real time qPCR primers** | |
| **Gene** | **Nucleotide sequence (5’-3’)** |
| Ythdc1 | Forward: GGGAATGATTATGACACCCGAAGT |
|  | Reverse: GGACAGCACGAACGGAAGATG |
| MyoG | Forward: ATGGTGCCCAGTGAATGCAA |
|  | Reverse: ACCCAGCCTGACAGACAATC |
| Acta1 | Forward: CACCAGGGTGTCATGGTAGG |
|  | Reverse: TGGTACGGCCGGAAGCATAG |
| Tnni2 | Forward: CCTGAAGAGTGTGATGCTCCA |
|  | Reverse: CCCGTTCCTTCTCAGTGTCTT |
| Myh3 | Forward: TATCAGAGTGAGGAGGACAG |
|  | Reverse: TCGCTTTCATGGACCACCAT |
| Gapdh | Forward: AGAACATCATCCCTGCATCC |
|  | Reverse: GGTCCTCAGTGTAGCCCAAG |
| **Nucleotide sequence of RT-PCR primers** | |
| **Gene** | **Nucleotide sequence (5’-3’)** |
| Pi4kb | Forward: ACTGAGCATGGAAACTGA |
|  | Reverse: TTATCCTTGGAATTGAGAA |
| Pi4k2a | Forward: TGCAAGCGAATCCTACAC |
|  | Reverse: CTCCTGATGGCAAACCTG |
| **Nucleotide sequence of RIP real-time qPCR primers** | |
| **Gene** | **Nucleotide sequence (5’-3’)** |
| Pi4kb | Forward: AGGGAGGAAGAAGATGAGA |
|  | Reverse: TGCGAAAGTAGAAGAGCC |
| Pi4k2a | Forward: AGACCAGGTTTGCCATCA |
|  | Reverse: CACTCCCAAGTCCACATTC |
